# Nitrogen Removal from Micro-Polluted Reservoir Water by Indigenous Aerobic Denitrifiers

**DOI:** 10.3390/ijms16048008

**Published:** 2015-04-10

**Authors:** Ting-Lin Huang, Shi-Lei Zhou, Hai-Han Zhang, Na Zhou, Lin Guo, Shi-Yu Di, Zi-Zhen Zhou

**Affiliations:** School of Environmental and Municipal Engineering, Xi’an University of Architecture and Technology, Xi’an 710055, China; E-Mails: ZSLZhouShilei@126.com (S.-L.Z.); zhanghaihan@xauat.edu.cn (H.-H.Z.); zhounastone@163.com (N.Z.); guolin9608@aliyun.com (L.G.); dishiyu0924@gmail.com (S.-Y.D.); zhouzizhen001@sina.com (Z.-Z.Z.)

**Keywords:** aerobic denitrifiers, aerobic denitrification, nitrogen removal, source water

## Abstract

Treatment of micro-polluted source water is receiving increasing attention because of environmental awareness on a global level. We isolated and identified aerobic denitrifying bacteria *Zoogloea* sp. N299, *Acinetobacter* sp. G107, and *Acinetobacter* sp. 81Y and used these to remediate samples of their native source water. We first domesticated the isolated strains in the source water, and the 48-h nitrate removal rates of strains N299, G107, and 81Y reached 33.69%, 28.28%, and 22.86%, respectively, with no nitrite accumulation. We then conducted a source-water remediation experiment and cultured the domesticated strains (each at a dry cell weight concentration of 0.4 ppm) together in a sample of source water at 20–26 °C and a dissolved oxygen concentration of 3–7 mg/L for 60 days. The nitrate concentration of the system decreased from 1.57 ± 0.02 to 0.42 ± 0.01 mg/L and that of a control system decreased from 1.63 ± 0.02 to 1.30 ± 0.01 mg/L, each with no nitrite accumulation. Total nitrogen of the bacterial system changed from 2.31 ± 0.12 to 1.09 ± 0.01 mg/L, while that of the control system changed from 2.51 ± 0.13 to 1.72 ± 0.06 mg/L. The densities of aerobic denitrification bacteria in the experimental and control systems ranged from 2.8 × 10^4^ to 2 × 10^7^ cfu/mL and from 7.75 × 10^3^ to 5.5 × 10^5^ cfu/mL, respectively. The permanganate index in the experimental and control systems decreased from 5.94 ± 0.12 to 3.10 ± 0.08 mg/L and from 6.02 ± 0.13 to 3.61 ± 0.11 mg/L, respectively, over the course of the experiment. Next, we supplemented samples of the experimental and control systems with additional bacteria or additional source water and cultivated the systems for another 35 days. The additional bacteria did little to improve the water quality. The additional source water provided supplemental carbon and brought the nitrate removal rate in the experimental system to 16.97%, while that in the control system reached only 3.01%, with no nitrite accumulation in either system. Our results show that aerobic denitrifying bacteria remain highly active after domestication and demonstrate the applicability of such organisms in the bioremediation of oligotrophic ecosystems.

## 1. Introduction

Economic and social development has resulted in massive amounts of nitrogenous compounds in environmental waters and led to the continuous deterioration of aquatic ecosystems [1,2,3,4,5] and eutrophication of some reservoirs [6,7], putting drinking water sources at great risk [5]. Many physical [8], chemical [9], and biological methods [10] have been used to remove the nitrogenous compounds and purify polluted waters [11]. Bioremediation has attracted wide attention because it has lower maintenance costs and greater pollutant-removal performance than other methods [10], for example, remediation of uranium [12], toluene [13,14], and organic and metal contamination [15]. Traditional denitrification occurs under anaerobic or anoxic conditions [16] through a sequence of intermediates (nitrate, nitrite, nitric oxide, and nitrous oxide), finally ending with nitrogen gas [17,18,19].

The reaction steps that use nitrate or nitrite as terminal electron acceptors are inhibited by oxygen [20]. *Paracoccus pantotrophus*, first isolated by Robertson and Kuenen [21] and later renamed as *Thiosphaera pantotropha*, is an aerobic denitrifier that has been studied to the greatest extent. There have been recent reports of aerobic denitrifying bacteria isolated from canals [22], soils [23], ponds [24], activated sludge [25], and lakes [26] that can simultaneously utilize oxygen and nitrate as electron acceptors. Most of the isolated strains, such as *T. pantotropha* [27], *Alcaligenes faecalis* [25,28,29], *Pseudomonas stutzeri* [22], *Pseudomonas putida* [23,30], *Pseudomonas mendocina* [24], *Pseudomonas aeruginosa* [31], *Citrobacter diversus* [32], and *Bacillus subtilis* [33,34], have been used to treat wastewater [25]. Patureau *et al.* [35] proposed a more integrated configuration for nitrogen and phosphorus removal through the introduction of an aerobic denitrifier into a complex phosphorus/nitrifying ecosystem. Barak *et al.* [36] concluded that *Pseudomonas denitrificans* is capable of combined phosphate and nitrate removal without the need for alternating anaerobic/aerobic or anaerobic/anoxic switches. Joo *et al.* [25] found that *P. denitrificans* removed more than 65% of the ammonia from polluted water and showed that one strain performed heterotrophic nitrification and aerobic denitrification in piggery wastewater.

However, these strains might have acclimation problems in eutrophic freshwater reservoirs because of the relatively low nitrate and ammonium concentrations and low carbon levels, which could limit the denitrification process [37]. Indigenous strains that can perform aerobic denitrification in micro-polluted reservoir water would help to overcome this problem.

Aerobic denitrifiers are rarely isolated from reservoirs [26,38], and there is little or no research to date on the use of aerobic denitrifiers to denitrify and bioremediate reservoir ecosystems. Several studies have illustrated the difficulties in removing nitrogen from source waters because of the relatively low concentrations of nitrogen as a pollutant [39,40]. We studied the effects of aerobic denitrifiers on water quality, and some of our previous findings have been reported elsewhere [41,42,43,44,45]. The objective of this study was to determine the phylogenetic affiliation of three strains of indigenous aerobic denitrifiers by using 16S rRNA sequencing and to assess the nitrogen removal performance of these strains in micro-polluted reservoir water.

## 2. Results

### 2.1. Identification and Phylogenetic Analysis of Three Aerobic Denitrifiers

**Figure 1:**
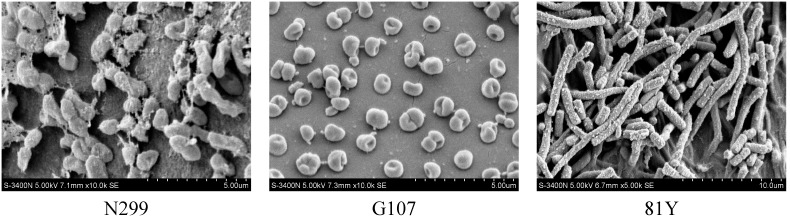
Scanning electron microscope images of the aerobic denitrifiers.

**Figure 2:**
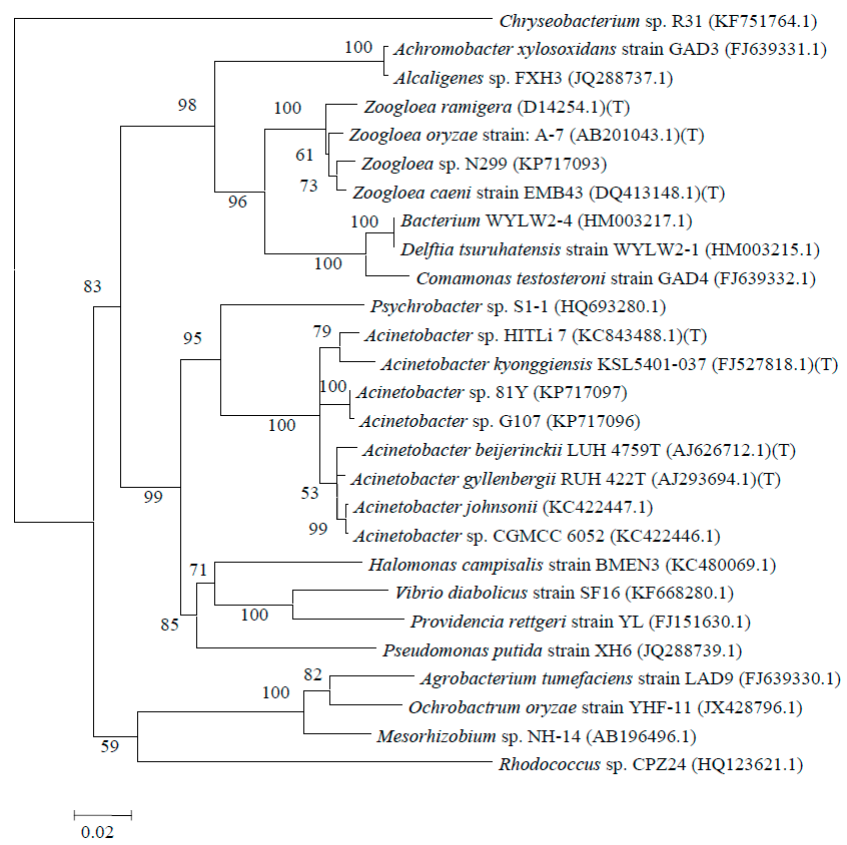
Phylogenetic tree based on the comparison of partial 16S rRNA gene sequences of strains N299, G107, and 81Y, other type culture strains, and other previously studied aerobic denitrifiers. The tree was constructed using the neighbour-joining method with bootstrap values of 1000 replications.

Three gram-negative, oligotrophic aerobic denitrifying bacterial strains were isolated, submitted to GenBank, and identified as *Zoogloea* sp. N299, *Acinetobacter* sp. G107, and *Acinetobacter* sp. 81Y (Table 1). The sizes of the three strains were approximately 0.5–1 μm × 1.5–3 μm, 0.5–1 μm × 1–1.5 μm, and 0.5–1 μm × 2–8 μm (Figure 1). Figure 2 shows the neighbor-joining phylogenetic tree prepared using partial 16S rRNA gene sequences from strains N299, G107, and 81Y, type culture strains and other previously studied aerobic denitrifiers. On the basis of the 16S rRNA gene sequences, strain N299 clustered with species from *Zoogloea* and strains G107 and 81Y clustered with species from *Acinetobacter*.

**Table 1:** 16S rRNA sequence similarities between the isolates and reference sequences.

Strains	GenBank No.	Sequence Length	Reference Sequences	Similarity (%)
81Y	KP717097	1315	*Acinetobacter pittii* CIP 70.29(T)	99.92
G107	KP717096	1392	*Acinetobacter pittii* CIP 70.29(T)	99.57
N299	KP717093	1361	*Zoogloea caeni* EMB43(T)	97.85

### 2.2. Gradient Domestication

**Figure 3:**
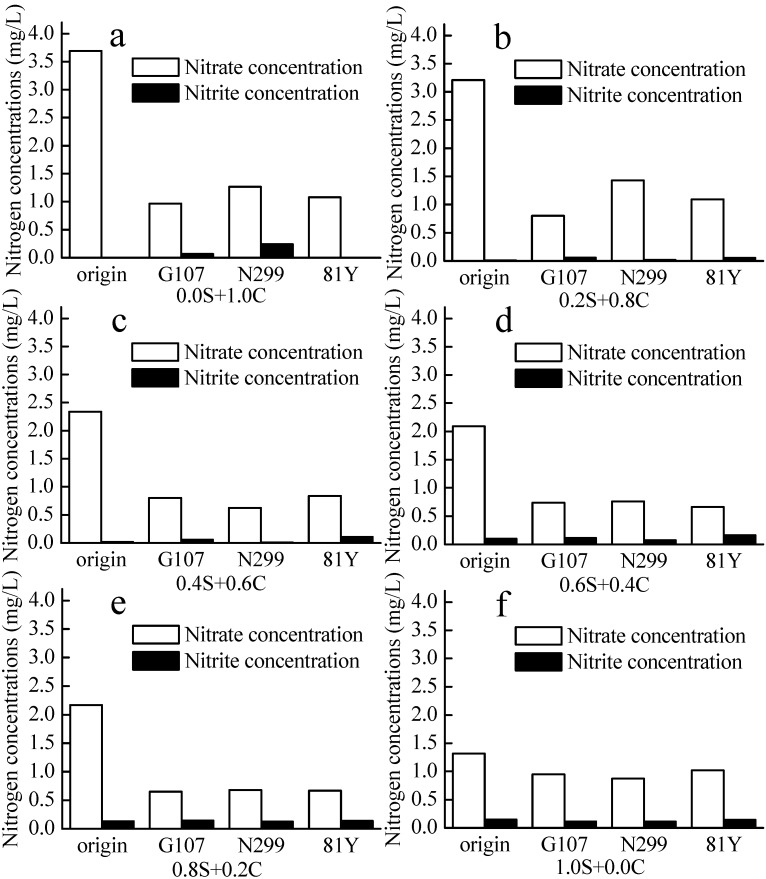
The nitrate and nitrite concentrations in domestication of the source water experiment. (**a**) 0.0 S + 1.0 C represents 0% source water + 100% culture medium; (**b**) 0.2 S + 0.8 C represents 20% source water + 80% culture medium; (**c**) 0.4 S + 0.6 C represents 40% source water + 60% culture medium; (**d**) 0.6 S + 0.4 C represents 60% source water + 40% culture medium; (**e**) 0.8 S + 0.2 C represents 80% source water + 20% culture medium; (**f**) 1.0 S + 0.0 C represents 100% source water + 0% culture medium.

**Figure 4:**
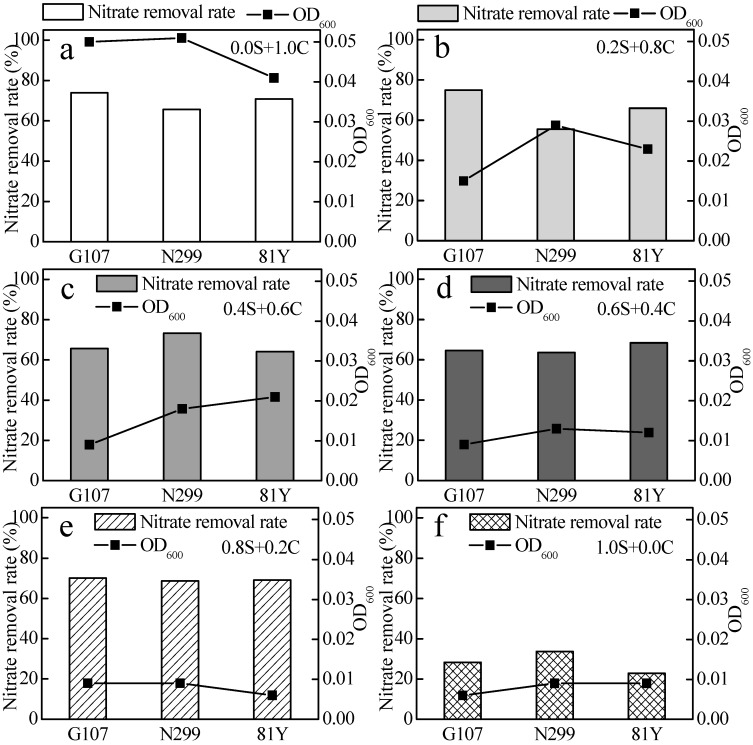
The nitrate removal and OD_600_ in domestication of the source water experiment. (**a**) 0.0 S+ 1.0 C represents 0% source water + 100% culture medium; (**b**) 0.2 S + 0.8 C represents 20% source water + 80% culture medium; (**c**) 0.4 S + 0.6 C represents 40% source water + 60% culture medium; (**d**) 0.6 S + 0.4 C represents 60% source water + 40% culture medium; (**e**) 0.8 S + 0.2 C represents 80% source water + 20% culture medium; (**f**) 1.0 S + 0.0 C represents 100% source water + 0% culture medium.

Strains N299, G107, and 81Y were adapted to the source water environment by gradient domestication, which lasted 12 days. The nitrate removal rates of N299, G107, and 81Y during the 100% source-water domestication experiment were 33.69%, 28.28%, and 22.86%, respectively, in 48 h, and no nitrite was accumulated in the cultures (Figure 3 and Figure 4). The densities of the three strains in the source water at the end of the domestication was 10^6~7^ cfu/mL, providing a good preparation for the subsequent experiments (Table 2).

**Table 2:** Indicators of bacterial densities in the 100% source water domestication cultures.

Strains	Colonies/Lg	SD	DCW/(mg/mL)	SD
N299	6.78	0.01	0.39	0.04
G107	6.41	0.07	0.48	0.06
81Y	6.76	0.03	0.42	0.06

SD: Standard deviation of three replicates; DCW: Dry cell weight of three replicates.

### 2.3. Dissolved Oxygen Concentration and Temperature in the Source Water Denitrification Experiment

**Figure 5:**
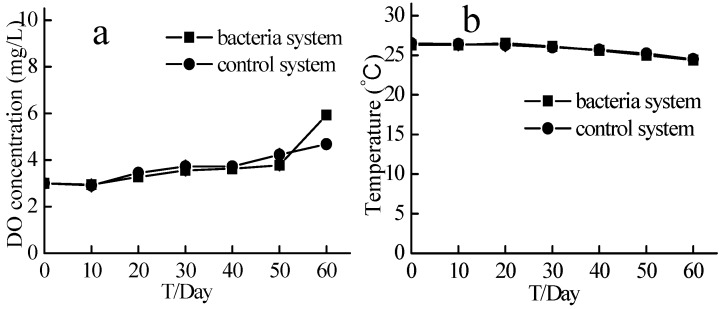
DO and Temperature of the source water experiment. Bacteria system respents system with aerobic denitrification bacteria N299, G107 and 81Y; Control system respents system without aerobic denitrification bacteria N299, G107 and 81Y; (**a**) DO concentration of bacteria and control system; (**b**) Temperature of bacteria and control system.

In the 60-day source water denitrification experiment, the temperature of the experimental system and the control system was 20–27 °C, and the dissolved oxygen concentration (DOC) of the two systems was maintained at 3–7 mg/L (Figure 5).

### 2.4. Nitrate and Total Nitrogen Concentrations in the Source Water Denitrification Experiment

**Figure 6:**
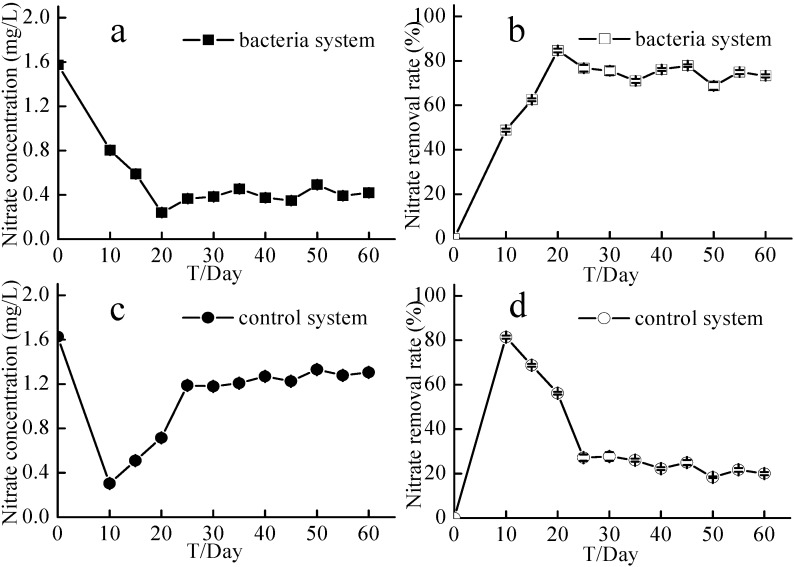
Nitrate concentrations and nitrate removal rates of the source water experiment. Bacteria system respents system with aerobic denitrification bacteria N299, G107 and 81Y; Control system respents system without aerobic denitrification bacteria N299, G107 and 81Y; (**a**) nitrate concentration of bacteria system; (**b**) nitrate removal rate of bacteria system; (**c**) nitrate concentration of control system; (**d**) nitrate removal rate of control system.

**Figure 7:**
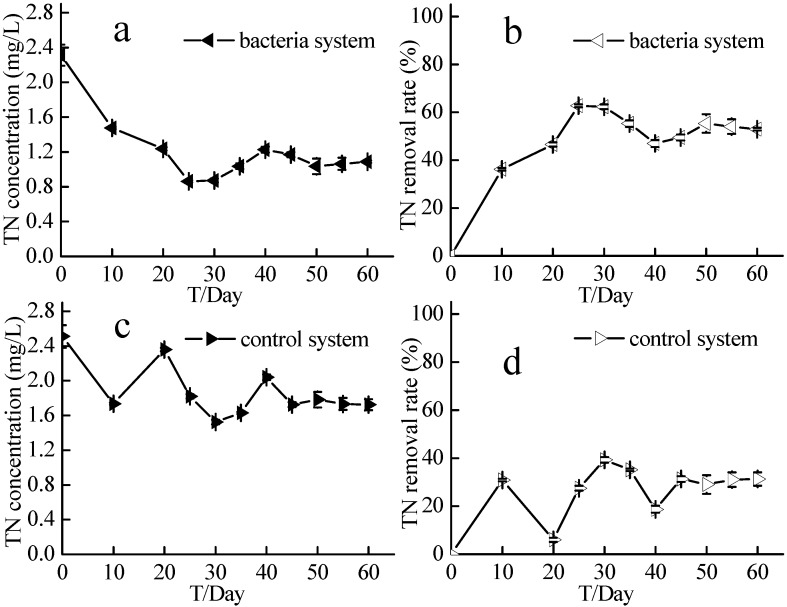
TN concentrations and TN removal rates of the source water experiment. Bacteria system respents system with aerobic denitrification bacteria N299, G107 and 81Y; Control system respents system without aerobic denitrification bacteria N299, G107 and 81Y; (**a**) TN concentration of bacteria system; (**b**) TN removal rate of bacteria system; (**c**) TN concentration of control system; (**d**) TN removal rate of control system.

Nitrate and total nitrogen (TN) concentrations in the source water in the experimental system and control system reached a stable state after 30 days, which lasted until the end of the 60-day experiment (Figure 6 and Figure 7). In the first 30 days, nitrate concentration in the experimental system decreased from 1.57 ± 0.02 to 0.38 ± 0.01 mg/L, while that in the control system decreased from 1.63 ± 0.02 to 1.18 ± 0.03 mg/L. At the end of the 60-day experiment, nitrate concentration in the experimental and control systems was 0.42 ± 0.01 and 1.30 ± 0.01 mg/L, respectively. During the first 30 days of the experiment, the nitrate removal rate in the experimental system was at least 70%, while that in the control system was around 20%. TN in the experimental system was significantly lower than that in the control system (Figure 7). At the end of the 60-day experiment, TN in the experimental system was 1.09 ± 0.01 mg/L, while that in the control system was 1.72 ± 0.06 mg/L. TN removal rate in the experimental system averaged about 50%, while that in the control system was 20%–30%.

### 2.5. Nitrite, Permanganate Index, and Bacterial Densities in the Source Water Denitrification Experiment

**Figure 8:**
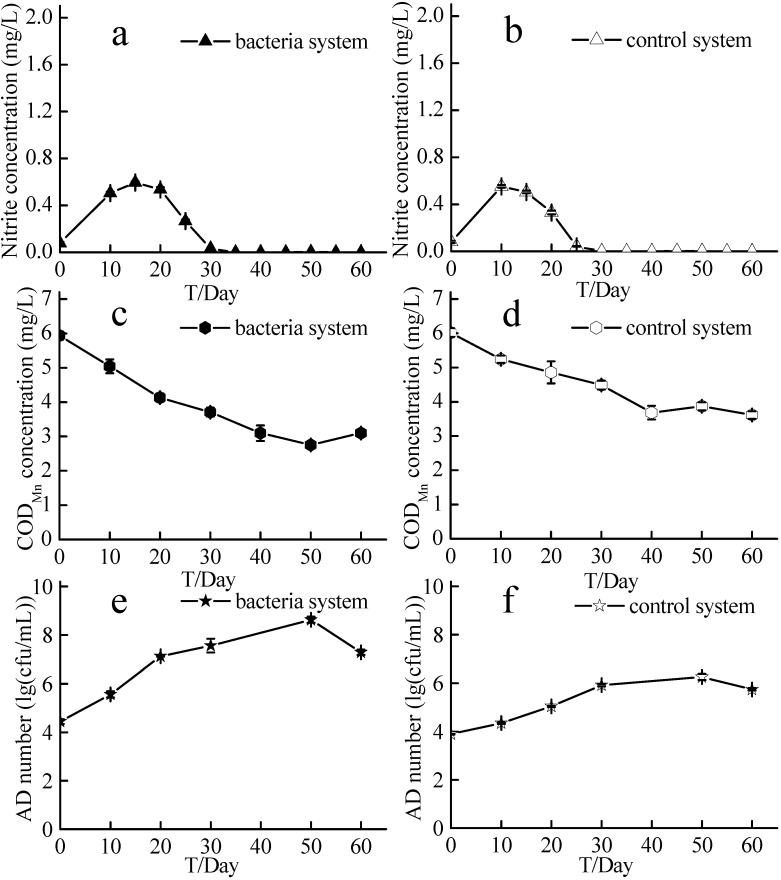
Nitrite concentrations, COD_Mn_, and the densities of aerobic denitrification bacteria of the source water experiment. AD number represents the densities of aerobic denitrification bacteria; Bacteria system represents the system with aerobic denitrification bacteria N299, G107 and 81Y; Control system means system without aerobic denitrification bacteria N299, G107 and 81Y; (**a**) nitrite concentration of bacteria system; (**b**) nitrite concentration of control system; (**c**) COD_Mn_ concentration of bacteria system; (**d**) COD_Mn_ concentration of control system; (**e**) AD number of bacteria system; (**f**) AD number of control system.

As shown in Figure 8, nitrite concentration in the experimental system increased from 0.07 ± 0.01 to 0.59 ± 0.01 mg/L in 15 days and then decreased to 0.03 ± 0.00 mg/L, while that in the control system increased from 0.08 ± 0.01 to 0.55 ± 0.01 mg/L and then decreased to 0 ± 0.00 mg/L. The permanganate index (COD_Mn_) in the experimental system decreased from 5.94 ± 0.12 to 3.10 ± 0.08 mg/L, while that in the control system decreased from 6.02 ± 0.13 to 3.61 ± 0.11 mg/L. The density of aerobic denitrifying bacteria in the experimental system was higher than that in the control system, particularly at day 50 when the density in the experimental system reached 4.36 × 10^8^ cfu/mL and that in the control system was 1.87 × 10^6^ cfu/mL.

### 2.6. Experiment with Supplemental Bacteria

The temperature of the experimental and control systems was 26–28 °C over the course of the experiment. At the end of the 35-day experiment, the system supplemented with additional bacteria contained 0.5 mg/L nitrate, 0 mg/L nitrite, and ~1.0 mg/L TN (Table 3 and Table 4). The control system contained ~1.5 mg/L nitrate at the end of the experiment, and the nitrate removal rate was less than 20%, which was consistent with the previous control system. There was no nitrite detected in the control system, and the TN removal rate was about 20%.

**Table 3:** The results of addition bacteria system.

Day	Nitrate (mg/L)	Nitrate Removal Rate (%)	Nitrite (mg/L)	TN (mg/L)	TN Removal Rate (%)	COD	DO (mg/L)	T (°C)
0	0.61	61.15	0	1.04	48.26	3.65	7.23	27.20
10	0.48	69.43	0	1.51	24.88	3.13	5.94	28.40
20	0.63	60.18	0.02	1.53	23.72	3.03	5.46	25.5
30	0.51	67.57	0.01	1.02	49.46	2.95	6.82	28.3
35	0.46	70.42	0	—	—	2.92	7.03	26.0

—, no detected.

**Table 4:** The results of the control system.

Day	Nitrate (mg/L)	Nitrate Removal Rate (%)	Nitrite (mg/L)	TN (mg/L)	TN Removal Rate (%)	COD	DO (mg/L)	T (°C)
0	1.41	13.50	0.001	1.61	35.86	3.83	6.50	26.20
10	1.28	21.47	0	2.56	−1.99	2.47	6.90	27.90
20	1.72	−5.76	0.01	2.31	7.99	2.00	8.18	27.50
30	1.40	13.97	0.04	1.85	26.31	2.25	7.27	27.70
35	1.72	−5.76	0	—	—	2.32	7.67	27.40

—, no detected.

### 2.7. Supplemental Carbon Experiment

**Figure 9:**
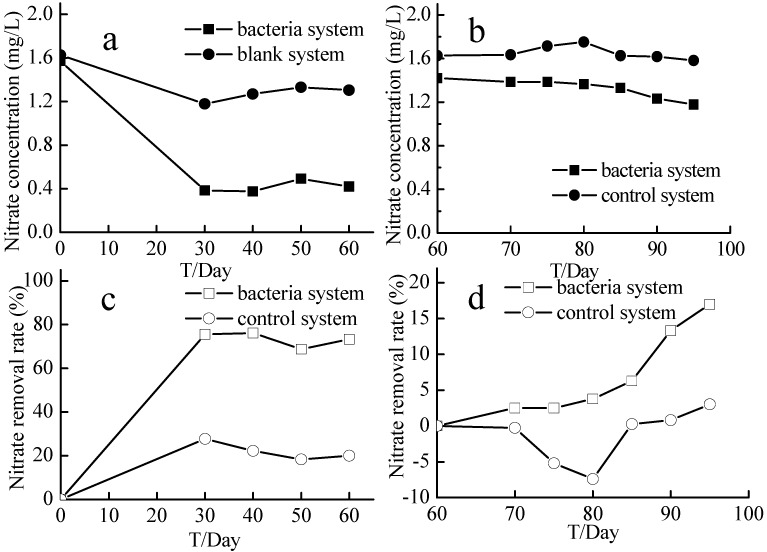
Nitrate concentrations and nitrate removal rates of the supplemental carbon experiment. Bacteria system means system with aerobic denitrification bacteria N299, G107 and 81Y; Control system means system without aerobic denitrification bacteria N299, G107 and 81Y; (**a**) nitrate concentration of bacteria and control system (0~60 days); (**b**) nitrate concentration of bacteria and control system (60~95 days); (**c**) nitrate removal rate of bacteria and control system (0~60 days); (**d**) nitrate removal rate of bacteria and control system (60~95 days).

**Figure 10:**
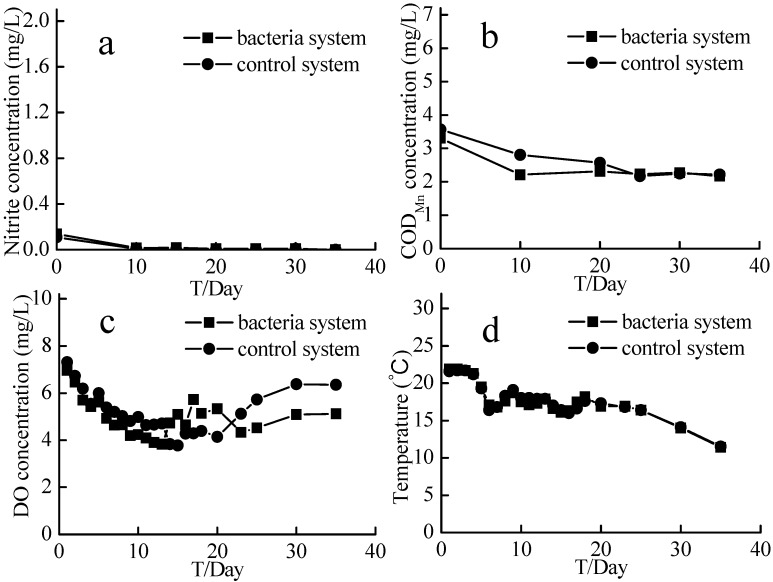
The nitrite, COD_Mn_, Temperature, and DO of the supplemental carbon experiment. Bacteria system represents system with aerobic denitrification bacteria N299, G107 and 81Y; Control system means system without aerobic denitrification bacteria N299, G107 and 81Y; (**a**) nitrite concentration of bacteria and control system; (**b**) COD_Mn_ concentration of bacteria and control system; (**c**) DO concentration of bacteria and control system; (**d**) Temperature of bacteria and control system.

Nitrate concentration in the experimental system dropped from 1.42–1.18 mg/L, while that in the control system dropped from 1.63–1.58 mg/L (which was essentially unchanged; Figure 9). At the end of the 35-day experiment, the nitrate removal rate in the experimental system was 16.97%, while that in the control system was 3.01%. After 10 days, nitrite levels in the two systems were both very low, at 0.02 mg/L or less (Figure 10a). The COD_Mn_ in the experimental system decreased from 3.3–2.17 mg/L, and that in the control system decreased from 3.57–2.22 mg/L (Figure 10b). The temperature of the cultures ranged from 22–11 °C, and the DOC was 3.8–7.3 mg/L (Figure 10c,d).

## 3. Discussion

Through enrichment and domestication of reservoir sediments, total organic carbon (TOC) of the final system was almost 13 mg/L. Then, the aerobic denitrifying bacteria were domesticated [45] by a series of transfers through the following media concentrations: 100% culture medium (SM medium, ~30 mg/L carbon source), 80% culture medium + 20% source water (~25.2 mg/L carbon source), 60% culture medium + 40% source water (~20.4 mg/L carbon source), 40% culture medium + 60% source water (~15.6 mg/L carbon source), 20% culture medium + 80% source water (~10.8 mg/L carbon source), and 100% source water (~6 mg/L carbon source), according to Kuznetaov *et al.* [46]. Kuznetaov *et al.* defined oligotrophic bacteria that could first survive in a medium with 1–15 mg/L carbon source. Therefore, the isolated aerobic denitrifiers were oligotrophic bacteria. The three aerobic denitrification bacteria, N299, G107, and 81Y, had good nitrogen removal ability and were used to remediate reservoir source water.

Through gradient domestication in source water, the aerobic denitrifiers could adapt to the source water environment. All three strains showed good denitrification characteristics, which was consistent with the results of our previous studies [45]. The nitrate removal rates of N299, G107, and 81Y were 33.69%, 28.28%, and 22.86%, respectively, in 48 h. Zhou *et al.* [45] showed that after 72 h of cultivation in raw water, the nitrate and total nitrogen removal rates of two strains of aerobic denitrifying bacteria were 30%–40% and 20%–25%, respectively. After domestication, the bacteria in our experiment reached densities of 10^6^ cfu/mL, which were then used to remediate source water.

Addition of the three aerobic denitrifying strains to the source water increased the rates of nitrite and nitrate reduction, indicating good denitrification performance. Because nitrite is an intermediate of nitrification and denitrification, changes in nitrite levels in the system reflect nitrogen cycling by the bacteria. The nitrate level decreased while the nitrite level increased in a short period in the control system; this indicated that there was a certain number of denitrifying bacteria in the system, which is consistent with the findings of Carter *et al.* [47]. However, the denitrification performance of the system containing the three domesticated denitrifying strains was superior to that of the control system. The nitrogen in the control system could not be converted into its gaseous form and, hence, could not be removed completely. It was only converted to nitrite and then oxidized to nitrate, and thus it maintained a stable state until the end of the experiment. Previous studies have shown that most aerobic denitrifiers are able to denitrify at a DOC of less than 3 mg/L [48]. The strains used in our study could tolerate a high DOC of 3–7 mg/L, which is consistent with the findings of our previous study [44].

We evaluated the effect of bacterial growth of the aerobic denitrifying bacteria by using plate counts [44]. In addition, the nitrogen removal performances of the bacteria and the control system reflected the effect of bacterial growth in the experiment. The density of aerobic denitrifying bacteria in the experimental system was significantly higher than that in the control system, indicating that the domesticated aerobic denitrifiers persisted in the source water environment and maintained their denitrification abilities. The TN removal rate in the experimental system averaged 50%, while that in the control system was 20%–30%. There were a certain number of denitrifying bacteria in the natural system, which is consistent with the findings of Carter *et al.* (in a sediment system) [47], Zhang *et al.* (in canal systems) [22], Kim *et al.* (in soil systems) [23], Zhu *et al.* (in pond systems) [24], Joo *et al.* (in an activated sludge system) [25], Guo *et al.* (in lake systems) [26], and Huang *et al.* (in a reservoir system) [44,45,49]. Therefore, the TN removal rate in the control system also reached 20%–30%. In addition, the COD removal rate was higher in the experimental system than in the control system. Liu *et al.* [26] showed that the TN decreased slightly when a carbon source was added to the source water at a C/N ratio of 4, mainly due to the exhaustion of the carbon source. In a similar study, Xu *et al.* [24] showed that the TN removal rate reached 85% in filtered source water. Wei *et al.* [41] reported a TN removal rate of ~40% in source water over 60 days. Xu *et al.* [24] examined the ability of an aerobic denitrifier (*Pseudomonas mendocina* 3–7) isolated from the Hua-jia-Chi Pond in China to remediate polluted drinking water by performing experiments in different modified DM media, although the strain was not tested in real source water. Overall, the results of our study and those of previous studies suggest that biological agents can remediate source water environments, reduce the environmental impacts of industry and society, and have enormously valuable applications.

The experiment with supplemental bacteria was designed to investigate whether the addition of bacteria could reduce the concentrations of nitrogen or other nutrients and further purify the water. Because of the lack of a carbon source in the system, addition of bacteria did not further remove contaminants and purify the source water. The supplemental carbon experiment was designed to investigate whether the aerobic denitrifying bacteria were still highly active by adding more source water, which simulated a real reservoir environment. The nitrate concentration in the system that received the supplemental carbon showed a significant downward trend, while that in the control system did not change. Because the temperature of the experiment was not controlled, the temperature decreased from 21.9–11.4 °C over the course of the experiment. The denitrification process was sensitive to the temperature, and the denitrification rate doubled with every 4 °C increase in temperature [50]. Temperature was an important factor, which could have affected the enzymatic activity of aerobic denitrification bacteria [51]. Because of the low temperature, the nitrogen removal rater was not obvious. However, the data showed that the previously added aerobic denitrifying bacteria were still active. The rate of COD_Mn_ degradation in the supplemental carbon system was faster than that in the control system, which indicated the heterotrophic characteristics of the flora.

## 4. Experimental Section

### 4.1. Samples

Surface sediments (depth = 0–10 cm) and water samples were collected from an oligotrophic reservoir ecosystem (location: 34°56'38.74''N, 117°41'14.13''E) in June 2011 by using a sterilized Petersen stainless steel grab sampler [49]. The samples were stored in black plastic bags at 4 °C and transferred to the Key Laboratory of Northwest Water Resource, Environment and Ecology, Xi’an University of Architecture and Technology (Xi’an, China).

### 4.2. Enrichment Cultures and Isolation of the Aerobic Denitrifiers

A 100-mL stored sediment sample was added to 700 mL of the enrichment denitrification broth (in g·L^−1^: 0.5 CH_3_COONa, 0.1 NaNO_3_, 0.1 K_2_HPO_4_·3H_2_O, 0.05 CaCl_2_, 0.05 MgCl_2_·6H_2_O; pH 7.2) [43,49]. Every three days, the medium concentration was reduced by 10% by removing some of the volume and replacing it with an equal volume of water. Enrichment and domestication of the aerobic denitrifiers lasted almost a month [51]. The enrichment sediment suspension was sampled via gradient dilution in triplicate. The gradient dilutions were as follows: 10^−1^ dilution (1 mL enrichment sludge suspension added to 9 mL sterile distilled water), 10^−2^ dilution (1 mL·10^−1^ dilution suspension added to 9 mL sterile distilled water), 10^−3^ dilution (1 mL·10^−2^ dilution suspension added to 9 mL sterile distilled water), 10^−4^ dilution (1 mL 10^−3^ dilution suspension added to 9 mL sterile distilled water), 10^−5^ dilution (1 mL 10^−4^ dilution suspension added to 9 mL sterile distilled water), 10^−6^ dilution (1 mL 10^−5^ dilution suspension added to 9 mL sterile distilled water), and 10^−7^ dilution (1 mL 10^−6^ dilution suspension added to 9 mL sterile distilled water). The diluents were streaked on a solid screening medium (in g·L^−1^: 0.1 CH_3_COONa, 0.02 NaNO_3_, 0.02 K_2_HPO_4_·3H_2_O, 0.01 CaCl_2_, 0.01 MgCl_2_·6H_2_O, 20 agar; pH 7.2) [41] and incubated at 30 °C. Prominent single colonies were harvested and subsequently cultivated in SM medium (in g·L^−1^: 0.1 CH_3_COONa, 0.02 NaNO_3_, 0.02 K_2_HPO_4_·3H_2_O, 0.01 CaCl_2_, 0.01 MgCl_2_·6H_2_O, pH 7.2) with NaNO_3_ as the sole nitrogen source in order to assess the performance of the aerobic denitrifying bacteria. Three isolated strains (N299, G107, and 81Y) with high nitrogen removal performance were obtained and stored on SM slants at 4 °C and on Glycerin SM at −20 °C.

### 4.3. Analysis of 16S rRNA Gene Sequences

We performed polymerase chain reaction (PCR) by using the following primers [52]: 7F 5'-CAGAGTTGATCCTGGCT-3' and 1540R 5'-AGGAGGTGATCCAGCCGCA-3'. The PCR mix consisted of 2.5 μL 5× buffer (with Mg^2+^), 0.5 μL template, 2.5 mM of each dNTP, 0.2 μL Taq DNA polymerase, and sterile nuclease-free water (total volume, 25 μL). The PCR was performed as follows: 4 min at 94 °C, followed by 30 cycles of denaturation at 94 °C for 45 s, annealing at 55 °C for 15 s, and extension at 72 °C for 1 min. After a final extension at 72 °C for 10 min, the reaction mixtures were held at 4 °C. We screened the sequences for homology with the sequences in GenBank by using BLAST (http://blast.ncbi.nlm.nih.gov/Blast.cgi). A phylogenetic tree was constructed with the MEGA 5.0 program using the neighbor-joining method with 1000 bootstrap replicates and the maximum composite likelihood model.

### 4.4. Domestication of the Aerobic Denitrifiers in the Source Water

The three isolated aerobic denitrifying bacteria were inoculated into 50 mL liquid SM medium in separate 100 mL Erlenmeyer flasks and cultured for 24 h at 30 °C and 120 rpm. The aerobic denitrifying bacteria were then domesticated [45] by a series of transfers through the following media concentrations: 100% culture medium (SM medium), 80% culture medium + 20% source water, 60% culture medium + 40% source water, 40% culture medium + 60% source water, 20% culture medium + 80% source water, and 100% source water. Each gradient domestication medium was sterilized at 121 °C for 30 min prior to inoculation. We cultured the strains in 150 mL liquid medium in 250-mL Erlenmeyer flasks. We inoculated new culture media by 10% volumetric dilution every 48 h. We measured the nitrate and nitrite concentrations of the media to evaluate the performance of domestication. At the end of domestication, we measured the growth ability of the strains by performing plate counts [41] after 5 days of growth at 30 °C in solid SM medium (in g·L^−1^: 0.1 CH_3_COONa, 0.02 NaNO_3_, 0.02 K_2_HPO_4_·3H_2_O, 0.01 CaCl_2_, 0.01 MgCl_2_·6H_2_O, 20 agar; pH 7.2). We determined the dry cell weight (DCW) of the cultures by weighing cell pellets.

### 4.5. The 60-Day Source Water Denitrification Experiment

The experiment was performed in a 20-L bottle with a black plastic bag on the outer wall to simulate the darkness of the reservoir environment. DOC of the system was controlled using an aeration pump (Songbao, Zongshan, China). The system was maintained at room temperature. The three strains (N299, G107, and 81Y) after gradient domestication were added to the experimental system at 0.4 ppm (2 mL/20 L). Nitrate, nitrite, TN, COD, and aerobic denitrifying bacterial colonies were measured. All parameters were measured in triplicate. Nitrate, nitrite, and TN were measured every 5 days, and COD_Mn_ and number of colonies were measured every 10 days.

### 4.6. Experiment with Supplemental Bacteria

At the end of the 60-day source water denitrification experiment, we supplemented samples of the systems with bacteria to investigate whether adding bacteria could further purify the water. We added 0.4 ppm each of the three domesticated denitrifying bacteria to 5-L samples of the water from the previous 60-day experiment and from the control system. The 5-L bottles were wrapped in black plastic bags to simulate the darkness of the reservoir environment and incubated at 30 °C for 35 days. DOC was not controlled. Nitrate and nitrite were measured every 5 days, and COD_Mn_ was measured every 10 days.

### 4.7. Supplemental Carbon Experiment

At the end of the 60-day source water denitrification experiment, we performed a supplemental carbon experiment to investigate whether the aerobic denitrifying bacteria were still highly active. We added 5 L of water from the 60-day experiment and 15 L of fresh source water to 20-L bottles wrapped in black plastic bags to simulate the darkness of the reservoir environment. Nitrate and nitrite and COD_Mn_ were measured to reflect the experimental results. Nitrate and nitrite were measured every 5 days, and COD_Mn_ was measured every 10 days.

### 4.8. Data Analysis

The optical density of the culture broth was measured at 600 nm (OD_600_) by using a spectrophotometer (DR6000; HACH Company, Loveland, CO, USA) [53]. The nitrite concentration was determined by using *N*-(1-naphthalene)-diaminoethane photometry [54]. The TN and nitrate concentrations were measured by using hydrochloric acid photometry [54]. COD_Mn_ was analyzed using the potassium permanganate method [44]. DCW was determined by weighing the cell pellet after 5-mL samples of the broth cultures (SM medium) were dried in an oven at 105 °C for 12 h [53]. We used a scanning electron microscope (S-3400N; Hitachi, Tokyo, Japan). The densities of aerobic denitrifying bacteria in the cultures were measured by plate counts [40]. DOC and temperature were measured using HQ30d (HACH Company, Loveland, CO, USA). The samples of nitrate and nitrite were filtered using a 0.45-μm cellulose-acetate filter to remove the bacteria.

## 5. Conclusions

Through enrichment and domestication, three oligotrophic aerobic denitrification bacteria were obtained. On the basis of their 16S rRNA sequences, the aerobic denitrifiers were identified as *Zoogloea* sp. N299, *Acinetobacter* sp. G107, and *Acinetobacter* sp. 81Y. During the source-water gradient domestication, the three strains gradually adapted to the source water environment, suggesting that we can optimize domestication strategies to improve source-water denitrification capacity and the ability to adapt to a source of water in order to bioremediate micro-polluted source water.

The bacteria displayed strong denitrification characteristics during the 60 days in the source water. The nitrate concentration in the source water in the experimental system declined by at least 70%, while that in the control system declined by only 20%. There was no accumulation of nitrite in the system. In the subsequent experiment in which additional bacteria were added, there was no apparent difference between the experimental and control systems because of the lack of carbon. In the extension experiment in which additional carbon-containing source water was added, the nitrate removal rate in the experimental system reached 16.97% after 35 days, while that in the control system reached only 3.01%; this indicated that some aerobic denitrifying bacteria still survived in the source water from the 60-day experiment and maintained strong denitrification capabilities. The results support further experiments to characterize the bioremedial potential of aerobic denitrifying bacteria in source water. In order to better simulate the reservoir environment, experiments under additional sediment conditions should be performed.

We used a series of domestication and source-water experiments to show the feasibility of using aerobic denitrifying bacteria to bioremediate micro-polluted source water, suggesting a new way to reduce the environmental pollution load.

## References

[1] Galloway J.N., Townsend A.R., Erisman J.W., Bekunda M., Cai Z., Freney J.R., Martinelli L.A., Seitzinger S.P., Sutton M.A. (2008). Transformation of the nitrogen cycle: Recent trends, questions, and potential solutions. Science.

[2] Duce R.A., LaRoche J., Altieri K., Arrigo K.R., Baker A.R., Capone D.G., Cornell S., Dentener F., Galloway J., Ganeshram R.S. (2008). Impacts of atmospheric anthropogenic nitrogen on the open ocean. Science.

[3] Tilman D., Cassman K.G., Matson P.A., Naylor R., Polasky S. (2002). Agricultural sustainability and intensive production practices. Nature.

[4] Camargo J.A., Alonso Á. (2006). Ecological and toxicological effects of inorganic nitrogen pollution in aquatic ecosystems: A global assessment. Environ. Int..

[5] Qin B.Q., Zhu G.W., Gao G., Zhang Y.L., Li W., Paerl H.W., Carmichael W.W. (2010). A drinking water crisis in Lake Taihu, China: Linkage to climatic variability and lake management. Environ. Manag..

[6] Jiang C.L., Zhu L.Q., Hu X.Q., Cheng J.Y., Xie M.H. (2011). Reasons and control of eutrophication in new reservoirs. Eutrophication: Causes, Consequences and Control.

[7] Cai Q., Hu Z. (2006). Studies on eutrophication problem and control strategy in the Three Gorges Reservoir. Acta Hydrobiol. Sin..

[8] Li L., Wang H.W., Lu J.H. (2006). Nitrogen removal using air stripping tower in urban wastewater treatment plant. China Water Wastewater.

[9] Huang H.M., Song Q.W., Wang W.J., Wu S.W., Dai J.K. (2012). Treatment of anaerobic digester effluents of nylon wastewater through chemical precipitation and a sequencing batch reactor process. J. Environ. Manag..

[10] Zhu G.B., Peng Y.Z., Li B.K., Guo J.H., Yang Q., Wang S.Y., Whitacre D. (2008). Biological removal of nitrogen from wastewater. Reviews of Environmental Contamination and Toxicology.

[11] Abdul-Rahman R., Tsuno H., Zainol N. (2002). Nitrogen nutrient removals from wastewater and river water. Water Sci. Technol..

[12] Finneran K.T., Housewright M.E., Lovley D.R. (2002). Multiple influences of nitrate on uranium solubility during bioremediation of uranium-contaminated subsurface sediments. Environ. Microbiol..

[13] Sun W.M., Cupples A.M. (2012). Diversity of five anaerobic toluene-degrading microbial communities investigated using stable isotope probing. Appl. Environ. Microbiol..

[14] Sun W.M., Sun X.X., Cupples A.M. (2014). Presence, diversity and enumeration of functional genes (bssA and bamA) relating to toluene degradation across a range of redox conditions and inoculum sources. Biodegradation.

[15] Lovley D.R. (1995). Bioremediation of organic and metal contaminants with dissimilatory metal reduction. J. Ind. Microbiol..

[16] Kaspar H.F. (1982). Nitrite reduction to nitrous oxide by propionibacteria: Detoxication mechanism. Arch. Microbiol..

[17] Waki M., Yasuda T., Yokoyama H., Hanajima D., Ogino A., Suzuki K., Yamagishi T., Suwa Y., Tanaka Y. (2009). Nitrogen removal by co-occurring methane oxidation, denitrification, aerobic ammonium oxidation, and anammox. Appl. Microbiol. Biotechnol..

[18] Adav S.S., Lee D.J., Lai J.Y. (2009). Biological nitrification-denitrification with alternating oxic and anoxic operations using aerobic granules. Appl. Microbiol. Biotechnol..

[19] Adav S.S., Lee D.J., Lai J. (2010). Enhanced biological denitrification of high concentration of nitrite with supplementary carbon source. Appl. Microbiol. Biotechnol..

[20] Van Rijn J., Tal Y., Schreier H.J. (2006). Denitrification in recirculating systems: Theory and applications. Aquac. Eng..

[21] Robertson L.A., Kuenen J.G. (1983). *Thiosphaera pantotropha* gen. nov. sp. nov., a facultatively anaerobic, facultatively autotrophic sulphur bacterium. J. Gen. Microbiol..

[22] Zhang D.Y., Li W.G., Huang X.F., Qin W., Liu M. (2013). Removal of ammonium in surface water at low temperature by a newly isolated *Microbacterium* sp. Strain SFA13. Bioresour. Technol..

[23] Kim M., Jeong S.Y., Yoon S.J., Cho S.J., Kim Y.H., Kim M.J., Ryu E.Y., Lee S.J. (2008). Aerobic denitrification of *Pseudomonas putida* AD-21 at different C/N ratios. J. Biosci. Bioeng..

[24] Zhu L., Ding W., Feng L.J., Dai X., Xu X.Y. (2012). Characteristics of an aerobic denitrifier that utilizes ammonium and nitrate simultaneously under the oligotrophic niche. Environ. Sci. Pollut. Res..

[25] Joo H.S., Hirai M., Shoda M. (2006). Piggery wastewater treatment using *Alcaligenes faecalis* strain No. 4 with heterotrophic nitrification and aerobic denitrification. Water Res..

[26] Guo L.Y., Chen Q.K., Fang F., Hu Z.X., Wu J., Miao A.J., Xiao L., Chen X.F., Yang L.Y. (2013). Application potential of a newly isolated indigenous aerobic denitrifier for nitrate and ammonium removal of eutrophic lake water. Bioresour. Technol..

[27] Su J.J., Liu B.Y., Liu C.Y. (2002). Comparison of aerobic denitrification under high oxygen atmosphere by *Thiosphaera pantotropha* ATCC 35512 and *Pseudomonas stutzeri* SU2 newly isolated from the activated sludge of a piggery wastewater treatment system. J. Appl. Microbiol..

[28] Joo H.S., Hirai M., Shoda M. (2005). Characteristics of ammonium removal by heterotrophic nitrification-aerobic denitrification by *Alcaligenes faecalis* No. 4. J. Biosci. Bioeng..

[29] Niel E.W.J., Braber K.J., Robertson L.A., Kuenen J.G. (1992). Heterotrophic nitrification and aerobic denitrification in *Alcaligenes faecalis* strain TUD. Antonie van Leeuwenhoek.

[30] Liu J.J., Wang P., Wang H. (2008). Study on denitrification characteristics of a heterotrophic nitrification-aerobic denitrifier. Res. Environ. Sci..

[31] Chen F., Xia Q., Ju L.K. (2006). Competition between oxygen and nitrate respirations in continuous culture of *Pseudomonas aeruginosa* performing aerobic denitrification. Biotechnol. Bioeng..

[32] Huang H.K., Tseng S.K. (2001). Nitrate reduction by Citrobacter diversus under aerobic environment. Appl. Microbiol. Biotechnol..

[33] Yang X.P., Wang S.M., Zhang D.W., Zhou L.X. (2011). Isolation and nitrogen removal characteristics of an aerobic heterotrophic nitrifying–denitrifying bacterium, *Bacillus subtilis* A1. Bioresour. Technol..

[34] Yu A.R., Li Y., Yu J. (2004). Denitrification of a newly isolated *Bacillus* strain W2 and its application in aquaculture. J. Microbiol..

[35] Patureau D., Helloin E., Rustrian E., Bouchez T., Delgenes J.P., Moletta R. (2001). Combined phosphate and nitrogen removal in a sequencing batch reactor using the aerobic denitrifier, *Microvirgula* aerodenitrificans. Water Res..

[36] Barak Y., van Rijn J. (2000). Atypical polyphosphate accumulation by the denitrifying bacterium Paracoccus denitrificans. Appl. Environ. Microbiol..

[37] Obaja D., Macé S., Mata-Alvarez J. (2005). Biological nutrient removal by a sequencing batch reactor (SBR) using an internal organic carbon source in digested piggery wastewater. Bioresour. Technol..

[38] Zhu L., Ding W., Feng L.J., Kong Y., Xu J., Xu X.Y. (2012). Isolation of aerobic denitrifiers and characterization for their potential application in the bioremediation of oligotrophic ecosystem. Bioresour. Technol..

[39] Heaton T., Talma A., Vogel J. (1983). Origin and history of nitrate in confined groundwater in the western Kalahari. J. Hydrol..

[40] Wilson G.B., Andrews J.N., Bath A.H. (1990). Dissolved gas evidence for denitrification in the Lincolnshire Limestone groundwaters, eastern England. J. Hydrol..

[41] Huang T.L., Wei W., Su J.F., Zhang H.H., Li N. (2012). Denitrification performance and microbial community structure of a combined WLA-OBCO system. PLoS ONE.

[42] Wei W., Huang T.L., Li N. (2012). Denitrification characteristics of *in-situ* biological inoculation under conditions of low temperature and poor nutrient. Water Technol..

[43] Huang T.L., Li N., Zhang H.H., Wang K., Liu T.T. (2013). Denitrification characters and safety of communities of cold tolerant oligotrophic and aerobic denitrifying bacteria. Chin. J. Environ. Eng..

[44] Huang T.L., Wei W., Wang C.Y., Huang Z., Su J.F., Li Z. (2012). Pilot research on micropollutants removal in the raw water by combined process of water-lifting aeration and oligotrophic biofilm. J. Chongqing Univ..

[45] Huang T.L., Zhou N., Zhang H.H., Di S.Y., Zhou S.L., Guo L. (2014). Isolation and identification of three oligotrophic aerobic denitrifying bacteria and denitrification characteristics. Chin. J. Environ. Eng..

[46] Kuznetsov S., Dubinina G., Lapteva N. (1979). Biology of oligotrophic bacteria. Annu. Rev. Microbiol..

[47] Carter J.P., Hsaio Y., Spiro S., Richardson D.J. (1995). Soil and sediment bacteria capable of aerobic nitrate respiration. Appl. Environ. Microbiol..

[48] Su J.J., Liu B.Y., Lin J., Yang C.P. (2001). Isolation of an aerobic denitrifying bacterial strain NS2 from the activated sludge of piggery wastewater treatment systems in Taiwan possessing denitrification under 92% oxygen atmosphere. J. Appl. Microbiol..

[49] Wei W., Huang T.L., Su J.F., Wang C.Y., Huang Z., Li N. (2010). Isolation and identification of an oligotrophic and aerobic denitrification and its denitrification characteristics. Ecol. Environ. Sci..

[50] Zaitsev G., Mettänen T., Langwaldt J. (2008). Removal of ammonium and nitrate from cold inorganic mine water by fixed-bed biofilm reactors. Miner. Eng..

[51] Wei W. (2011). Properties and Experiments of Enhanced *in-situ* Biological Nitrogen Removal by Lifting Water and Aeration for Micro-Polluted Raw Water. Ph.D. Thesis.

[52] Tan L., Zhang X.Y., Cao T.L., Gai D.Y., Tian X.D. (2012). Isolation and identification of a new strain acidophilic heterotrophic bacteria from stone coal drainage. Adv. Mater. Res..

[53] Kim J.K., Park K.J., Cho K.S., Nam S.W., Park T.J., Bajpai R. (2005). Aerobic nitrification-denitrification by heterotrophic Bacillus strains. Bioresour. Technol..

[54] Chen X.F., Yang L.Y., Xiao L., Miao A.J., Xi B.D. (2012). Nitrogen removal by denitrification during cyanobacterial bloom in Lake Taihu. J. Freshw. Ecol..

